# Biomechanical changes during abdominal aortic aneurysm growth

**DOI:** 10.1371/journal.pone.0187421

**Published:** 2017-11-07

**Authors:** Raoul R. F. Stevens, Andrii Grytsan, Jacopo Biasetti, Joy Roy, Moritz Lindquist Liljeqvist, T. Christian Gasser

**Affiliations:** 1 Department of Biomedical Engineering, University of Technology, Eindhoven, The Netherlands; 2 Department of Biomedical Engineering, Maastricht University, Maastricht, The Netherlands; 3 KTH Solid Mechanics, School of Engineering Sciences, KTH Royal Institute of Technology, Stockholm, Sweden; 4 Department of Mechanical Engineering, Johns Hopkins University, Baltimore, United States of America; 5 Department of Molecular Medicine and Surgery, Karolinska Institute, Stockholm, Sweden; University of Zaragoza, SPAIN

## Abstract

The biomechanics-based Abdominal Aortic Aneurysm (AAA) rupture risk assessment has gained considerable scientific and clinical momentum. However, such studies have mainly focused on information at a single time point, and little is known about how AAA properties change over time. Consequently, the present study explored how geometry, wall stress-related and blood flow-related biomechanical properties change during AAA expansion. Four patients with a total of 23 Computed Tomography-Angiography (CT-A) scans at different time points were analyzed. At each time point, patient-specific properties were extracted from (i) the reconstructed geometry, (ii) the computed wall stress at Mean Arterial Pressure (MAP), and (iii) the computed blood flow velocity at standardized inflow and outflow conditions. Testing correlations between these parameters identified several nonintuitive dependencies. Most interestingly, the Peak Wall Rupture Index (PWRI) and the maximum Wall Shear Stress (WSS) independently predicted AAA volume growth. Similarly, Intra-luminal Thrombus (ILT) volume growth depended on both the maximum WSS and the ILT volume itself. In addition, ILT volume, ILT volume growth, and maximum ILT layer thickness correlated with PWRI as well as AAA volume growth. Consequently, a large ILT volume as well as fast increase of ILT volume over time may be a risk factor for AAA rupture. However, tailored clinical studies would be required to test this hypothesis and to clarify whether monitoring ILT development has any clinical benefit.

## Introduction

Degradation of elastin, collagen and apoptosis of Smooth Muscle Cells (SMC) [1] may lead to Abdominal Aortic Aneurysm (AAA) formation in the infrarenal aorta, which in turn may result in aortic rupture. Elective surgical or endovascular AAA repair is offered to prevent such catastrophic events, and repair is indicated as soon as the risk of aortic rupture exceeds the interventional risks. While the risks of intervention are reasonably predictable, assessing AAA rupture risk remains challenging during clinical decision making. Present clinical guidelines recommend AAA repair as soon as the diameter reaches 55mm or grows faster than 10mm/year [2,3], and diameter remains the most important surrogate marker of AAA risk [4]. However, a considerable portion of AAAs rupture below the size of 55mm (especially in female patients and current smokers [5]), whereas many aneurysms larger than 55mm never rupture [6–8]. Consequently, a more robust AAA rupture risk assessment would be of great clinical value.

The Biomechanical Rupture Risk Assessment (BRRA) quantitatively integrates many known risk factors for AAA rupture, allowing a more holistic risk assessment. The BRRA has gained considerable momentum [9–18], but the derived indices are essentially based on information at a single time point, and currently little is known about how AAA biomechanical parameters change over time.

Almost all clinically relevant AAAs contain an intra-luminal thrombus (ILT) [19] composed of fibrin and blood cells. The role of ILT is still contentious, but it is thought to play an important role in AAA progression. Despite ILT tissue being several times softer than the AAA wall, it may be large in volume, and thus having a significant structural impact on AAA biomechanics. Numerical [20,21] and in-vitro experimental [22] studies reported ILT’s structural impact, and the location of Peak Wall Stress (PWS) has been associated with the site of smallest ILT layer thickness [23]. Consequently, a thrombus layer may protect the vessel wall from rupture by acting as a stress buffer [20,22], thus decreasing the rupture risk of the aneurysm. However, when growing too thick, the ILT layer can cause the wall to weaken, for example due to hypoxia [24]. The ILT also provides an ideal environment for proteolytic agents [25]. These chemicals can be conveyed through the porous ILT [26,27] and diminish wall strength by proteolytic degradation of elastin and collagen. Such a wall weakening mechanism could explain why a thick ILT layer [28] and fast increase in ILT volume [29] have been linked to AAA rupture risk. A recent CT-A-based study [30] reported some consequences for AAA growth that might be linked to both aforementioned (competing) ILT-based mechanisms. The study found slowest AAA wall expansion behind an about seven millimeter thick ILT layer, i.e. ILT-based stress buffering seems to be fully compensated by ILT-based wall weakening once the ILT layer reached this thickness.

The present study aims at investigating how geometry, wall stress-related and blood flow-related biomechanical properties change during AAA expansion. Despite the fact that effects of blood flow on AAA growth have been reported [31], the interaction between these factors is still poorly understood. Knowledge about the time course of such parameters may lead to a better estimate of AAA rupture risk and improve monitoring protocols of AAA patients.

## Materials and methods

### Patient cohort

The use of anonymized patient data was approved by the Karolinska Institute ethics committee. AAA patients from Karolinska University Hospital, Stockholm, Sweden with at least five high resolution Computed Tomography-Angiography (CT-A) scan recordings within the last 10 years were included. Most of the CT-A scans were performed for diagnostic purposes and AAA surveillance. Patient characteristics are listed in Table 1. To avoid temporal fluctuations, the blood pressure was averaged over all available measurements.

**Table 1:** Patient characteristics and timeline of Computed Tomography-Angiography (CT-A) scans.

Patient ID	Age in years at baseline	Gender	Blood pressure (mmHg)	Number of CT-A scans (n) and follow-up times in years from baseline
**A**	76	male	140/80	(5) 0/0.7/2.2/2.7/3.9
**B**	64	female	207/113	(5) 0/2.0/3.0/4.0/5.9
**C**	63	male	160/100	(7) 0/0.6/1.5/2.7/4.2/5.3/8.4
**D**	73	female	140/80	(6) 0/0.3/0.6/1.3/3.5/3.7

### Geometrical analysis

**Fig 1:**
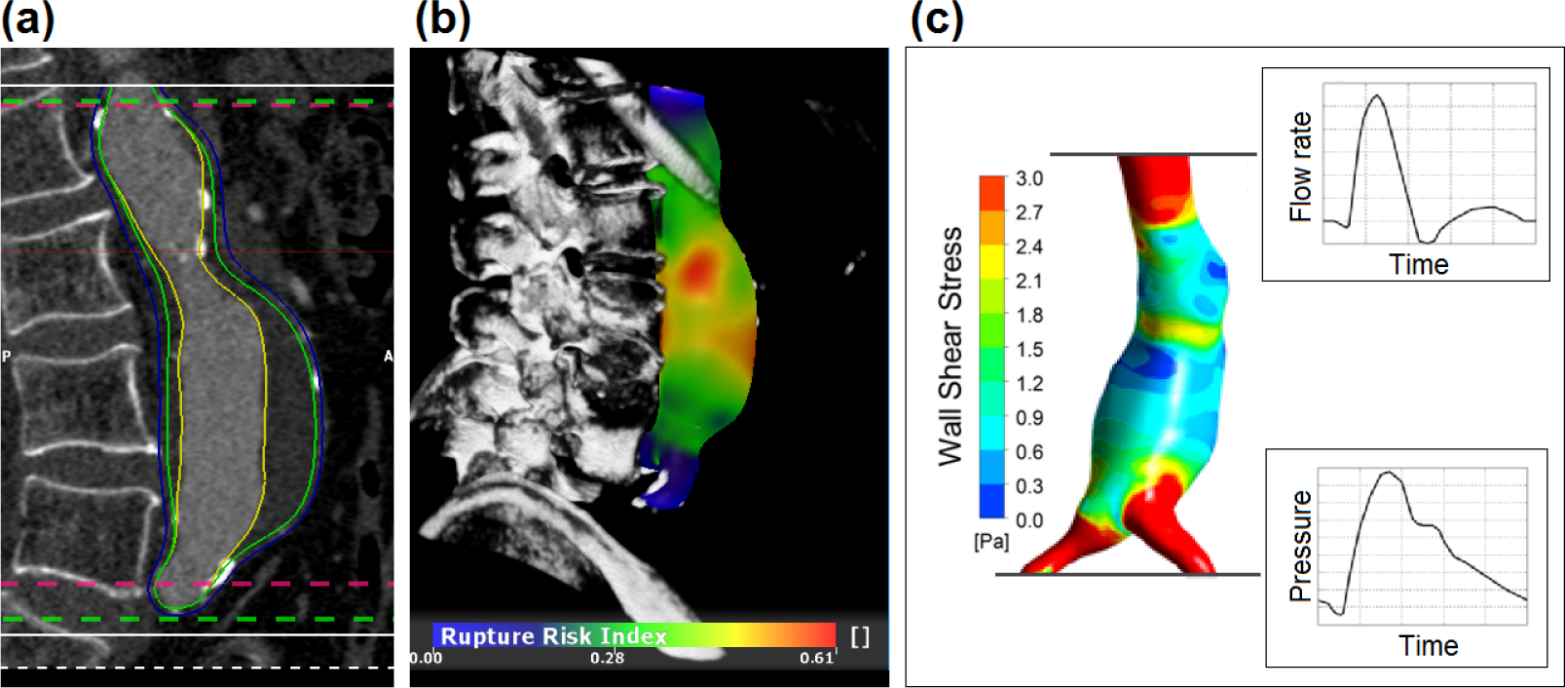
Analysis method performed for each patient at each time point. (a) Lateral Computed Tomography-Angiography (CT-A) slice with segmented Abdominal Aortic Aneurysm (AAA). Yellow, blue and green curves denote the luminal surface, exterior surface and wall-thrombus interface, respectively. (b) Rupture risk index plot derived from the structural biomechanics-based analysis at Mean Arterial Pressure (MAP). (c) Wall Shear Stress distribution at t = 0.25 s of the cardiac cycle derived from a Computational Fluid Dynamics (CFD) computation. At the inlet and the outlets, the indicated volume flow rate and pressure versus time responses were prescribed [36,37].

The aorta was semi-automatically segmented between the renal arteries and the aortic bifurcation (A4clinics Research Edition, VASCOPS GmbH, Graz, Austria). Segmented geometries included luminal and exterior AAA surfaces and used a predefined wall thickness that accounted for the reported wall thinning behind the ILT [28]. Specifically, in order to account for a moderate wall thinning behind the ILT layer, the wall thickness was set to $H_{WALL}=\max\;\left[1.5-\frac{0.17}{25}H_{ILT}\right]\;mm$ with *H*_ILT_ denoting the local thickness of the ILT layer in millimeters. Such predefined value compares reasonably to 1.56mm, an average value reported in the literature, see Table 2 in another study [32]. The reproducibility of the applied method has been reported previously [33–35], and a typical AAA segmentation is shown in Fig 1A. The maximum diameter (*d*_max_), the maximum ILT layer thickness (*H*_ILT max_), and luminal (*V*_lum_), thrombus (*V*_ILT_) and total (*V*_tot_) volumes were calculated for each aortic geometry. See Table 2 for further details.

**Table 2:** Definition of geometrical and biomechanical parameters. Bold face notation denotes vector or tensor quantities, and the region of interest was (manually) specified between the lower level of the renal arteries and the upper level of the aortic bifurcation, respectively.

Notation	Description	Remark
*Geometrical parameters*		
*d*_max_	Maximum outer diameter perpendicular to the luminal centerline	
*H*_ILT max_	Maximum thickness of the Intra-Luminal Thrombus (ILT) layer, i.e. maximum distance between wall-ILT interface and the luminal surface	
*V*_lum_,*V*_ILT,_*V*_tot_	Volumes of the lumen, ILT and the total vessel.	
*Structural biomechanical parameters*		
*PWS*	Peak Wall Stress. Highest von Mises stress in the wall all over the AAA	*PWS* = max[Wall stress]
*PWRI*	Peak Wall Rupture Index. Highest ratio between the calculated wall stress and the estimated wall strength all over the AAA.	$PWRI=max\left[\frac{Wall\;stress}{Wall\;strength}\right]$
*Hemodynamic biomechanical parameters*		
*v*_min_,*v*_max_,*v*_mean_	Minimal, maximal and mean magnitude of the blood flow velocity. The mean blood flow velocity is derived by averaging the magnitude of the blood flow velocity ***v*** over the time *T* of the cardiac cycle, as well as the volume of the lumen *V*_lum_	*v*_min_ = min[|***v***|]; *v*_max_ = max[|***v***|]; $v_{mean}=\frac{1}{T}\int_{0}^{T}\left[\frac{1}{V_{lum}}\int_{0}^{V_{lum}}\left|v\right|\;dv\right]\;dt$
${\dot{\gamma }}_{min},{\dot{\gamma }}_{max}$	Minimal and maximal scalar shear rates over the cardiac cycle. These quantities are derived from the spatial velocity gradient grad***v***, i.e. a quantity that denotes how fast the blood velocity changes in space.	${\dot{\gamma }}_{min}=\min\left[\dot{\gamma }\right];\;{\dot{\gamma }}_{max}=max[\dot{\gamma }]$ with $\dot{\gamma }=\sqrt{2l_{sym}l_{sym}}$ and ***l***_sym_ = (grad***v*** + grad^T^***v***)/2
WSS_min_,WSS_max_	Minimal and maximal magnitude of the Wall Shear Stress (WSS) vector ***WSS*** over the cardiac cycle. ***WSS*** denotes the mechanical stress induced by blood flow onto blood-tissue (wall or ILT) interface.	WSS_min_ = min[|***WSS***|] WSS_max_ = max[|***WSS***|]
*OSI*	Oscillatory Shear Index. The *OSI* is computed from the averaged magnitude of ***WSS*** and its magnitude |***WSS***|. The *OSI* denotes oscillatory behavior of the flow caused by complex flow patterns. Specifically, the extreme cases *OSI* = 1 and *OSI* = 0 denote oscillating and uni-directional flows, respectively.	$OSI=\frac{1}{2}\left(1-\frac{\left|AWSSV\right|}{AWSS}\right)$ with $AWSS=\frac{1}{T}\int_{0}^{T}\left|WSS\right|\;dt$ and $AWSSV=\frac{1}{T}\int_{0}^{T}WSS\;dt$

### Structural analysis

Non-linear Finite Element (FE) models were used to compute the stress in the AAA wall at Mean Arterial Pressure (MAP). Peak Wall Stress (PWS), i.e. the highest von Mises stress in the aneurysm wall, was extracted from each simulation (A4clinics Research Edition, VASCOPS GmbH, Graz, Austria). The FE model used hexahedral-dominated finite elements of Q1P0 formulation [38] to avoid volume locking of incompressible solids. The AAA was fixed at the renal arteries and at the aortic bifurcation, and no contact with surrounding organs was considered. Isotropic constitutive descriptions for the aneurysm wall [39] and the ILT [27] were assigned to each model with the ILT stiffness gradually decreasing from the luminal to the abluminal sites [27]. Specifically, the AAA wall was assumed to be homogenous and modeled by the two-parameter Yeoh strain energy function *ψ* = *c*_1_(*I*_1_ − 3) + *c*_2_(*I*_1_ − 3)^2^ with *I*_1_ = *tr* **C** denoting the first invariant of the right Cauchy-Green strain **C**. Here, the material parameters *c*_1_ = 77 *kPa* and *c*_2_ = 1881 *kPa* have been used, i.e. values identified from in-vitro AAA wall testing [39]. The ILT was modeled by an Ogden-type strain energy function $\psi =c\sum_{i=1}^{3}\left(\lambda_{i}^{4}-1\right)$ with *λ*_*i*_, *i* = 1,2,3 denoting the principal stretches. The constitutive properties of the ILT are captured by $c=\max\left[2.62-\frac{0.89}{25}H_{ILT},1.73\right]\;kPa$ with *H*_ILT_ denoting the local thickness of the ILT layer in millimeters. This expression accounts for the gradual decrease of stiffness from the luminal to the abluminal layer, i.e. as reported from in-vitro testing of ILT tissue [27]. The wall-ILT interface was rigid, i.e. ILT and AAA wall displacements matched at their interface.

A wall rupture risk index was defined by locally dividing the von Mises wall stress to an estimate of wall strength. AAA wall strength was assigned inhomogeneously and estimated by a scaled version [18,34] of the strength model proposed previously [12]. Finally, the highest wall risk index, or Peak Wall Rupture Index (*PWRI*), was extracted. In order to avoid picking up PWRI artefacts, A4clinics Research Edition averages over a sufficiently large number of FE nodes, i.e. locations where the wall rupture risk index is computed. In addition, PWRI location is indicated in the software window, so that the user can disregard identified artefacts. Fig 1B illustrates the typical distribution of the wall rupture risk index, and Table 2 details the investigated structural biomechanical parameters.

### Hemodynamical analysis

Rigid wall Computational Fluid Dynamics (CFD) models (ANSYS CFX, ANSYS Inc. US) with reported inflow and outflow conditions [36,37] were used to predict the blood flow velocity. Specifically, at the inlet, a plug velocity profile was derived from the inflow volume rate, and at both outlets, the pre-defined pressure was used. Inflow volume rate and outlet pressure wave have been taken from the literature [37]. The no-slip boundary condition was prescribed all along the luminal surface. The AAA lumen was meshed with tetrahedral finite volume elements (about 2mm in size), and five layers of prism elements (layer thickness ranging from 0.1mm to 0.2mm) aimed at capture boundary layer flow. Estimates on the required mesh size were based on our previous CFD work [36]. Specifically, a mesh sensitivity analysis [40] compared velocity, pressure, and WSS at ten points, to assess the relation between discretization error and element size.

The continuity and momentum equation were solved within the segment of the vascular lumen that has been segmented from CT-A images; in total five cardiac cycles with blood of density $\rho =1050\frac{kg}{m^{3}}$ were simulated. In addition, blood’s shear-thinning viscous properties were captured by the Carreau-Yasuda viscosity model $\mu =\mu_{\infty }+(\mu_{0}-\mu_{\infty }){\left[1+{\left(\lambda \dot{\gamma }\right)}^{\alpha }\right]}^{\frac{n-1}{\alpha }}$. Here, $\dot{\gamma }$ denotes the scalar shear rate, and *μ*_0_ = 0.16 *Pa s* and *μ*_∞_ = 0.0035 *Pa s* specified blood viscosity at low and high shear rates, respectively. In addition, the time constant *λ* = 8.2 *s*, the power law index *n* = 0.2128, and the Yasuda exponent *a* = 0.64 have been used. These parameters represent blood viscosity of blood at 37 degrees Celsius, and have been used previously [41,42]. Further details regarding the applied CFD, especially regarding verifying the plausibility of the predictions, are given elsewhere [43].

Hemodynamics parameters were extracted from the fifth calculated cardiac cycle and inside the aneurysmatic vessel domain (MATLAB, The MathWorks Inc., Natick, Massachusetts, USA). Specifically, the minimal (*v*_min_), maximal (*v*_max_) and mean (*v*_mean_) blood flow velocities, minimal (${\dot{\gamma }}_{min}$) and maximal (${\dot{\gamma }}_{max}$) scalar shear rates, minimal (*WSS*_min_) and maximal (*WSS*_max_) Wall Shear Stresses (WSS), as well as the Oscillatory Shear Index (*OSI*) [28,44] were computed. The definition of these parameters is listed in Table 2, and Fig 1C illustrates a typical *WSS* distribution, for example.

### Data analysis

Data analysis of biomechanical parameters was carried out within the aneurysmatic portions of the aorta. The proximal border of the aneurysmatic domain was defined by the vessel section at which the aorta was at least 10% larger than the normal (non aneurysmatic) aorta. The distal border was set 2.0cm proximal to the aortic bifurcation.

The rates of change over time of the geometrical, structural and hemodynamical were also investigated. At given time point, such quantities were calculated as the arithmetic difference between two consecutive CT-A scans and divided by the time between the scans. The rate of change of parameter X was denoted by ΔX.

Pooled data from all patients were statistically analyzed (SPSS, IBM Corp. Released 2013. IBM SPSS Statistics, Armonk, USA). All parameters were tested for normality using the Shapiro-Wilk test (significance level: *p* < 0.05), and Pearson and Spearman’s correlation tests (significance level: *p* < 0.05) were used to investigate simple correlation among normal and non-normal distributed parameters, respectively. Analysis of variance (ANOVA) was used to assess the statistical significance of multivariate linear regressions.

## Results

**Fig 2:**
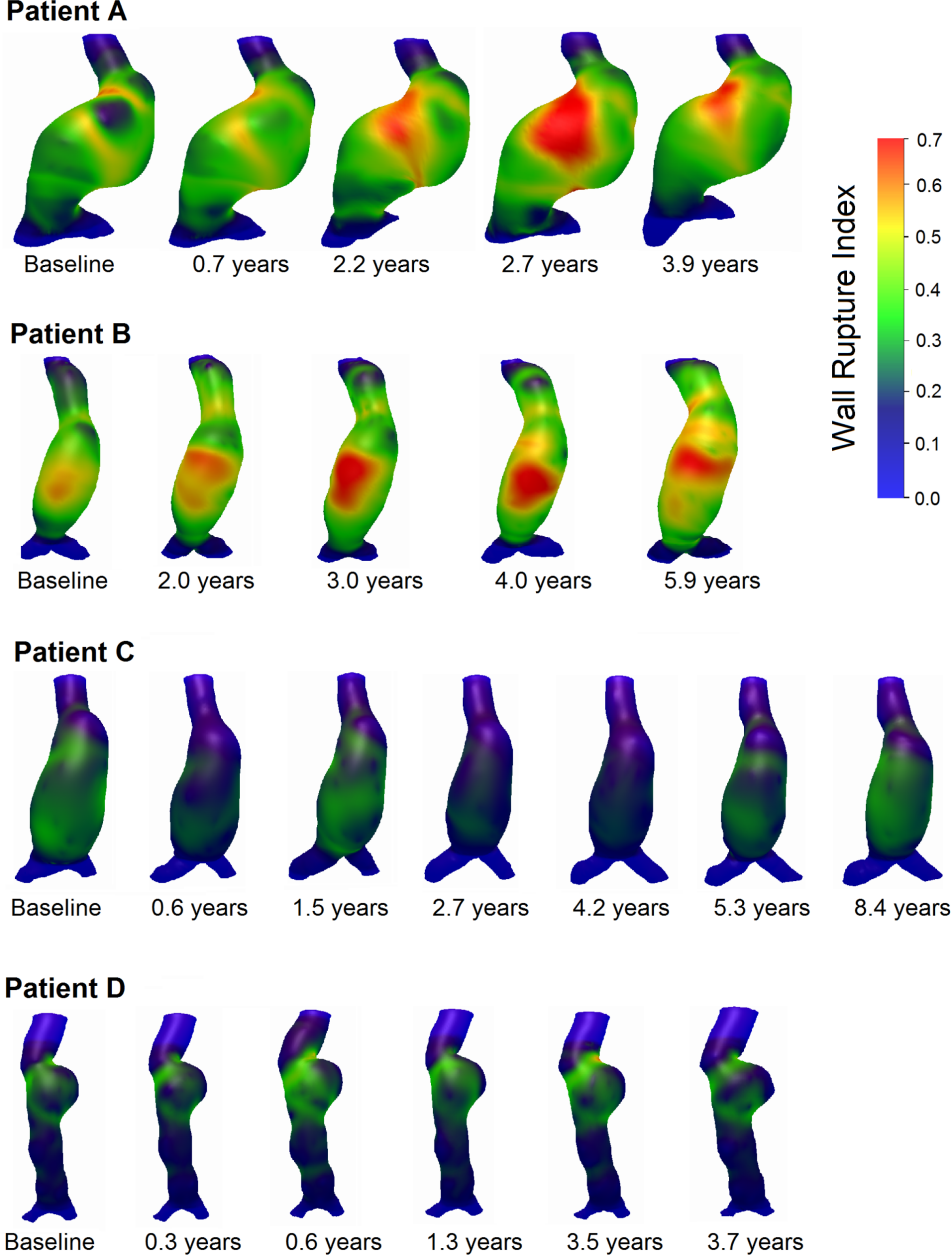
Development over time of the wall rupture risk index at Mean Arterial Pressure (MAP) in all four Abdominal Aortic Aneurysm (AAA) patients.

**Fig 3:**
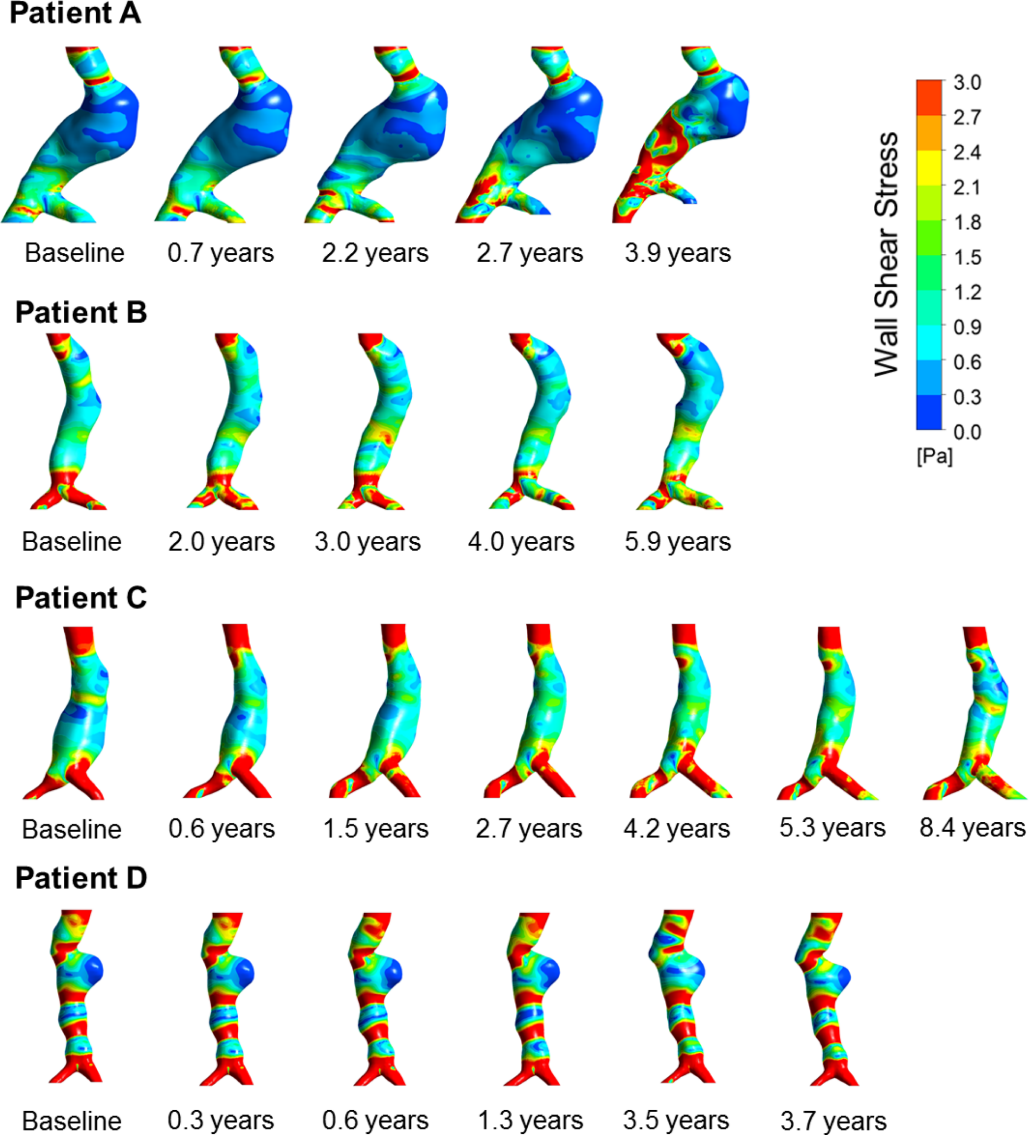
Development over time of the Wall Shear Stress (WSS) at t = 0.25 s of the cardiac cycle, i.e. at the time of peak blood inflow, in all four Abdominal Aortic Aneurysm (AAA) patients. Note that this time point does not correlate with the time when WSS peaks within the aneurysmatic portion of the aorta.

A complete analysis of a single case at one time point took about ten hours. Figs 2 and 3 illustrate the development of the wall rupture risk index and WSS over time for all four patients, respectively.

### Diameter and biomechanical rupture risk index

**Fig 4:**
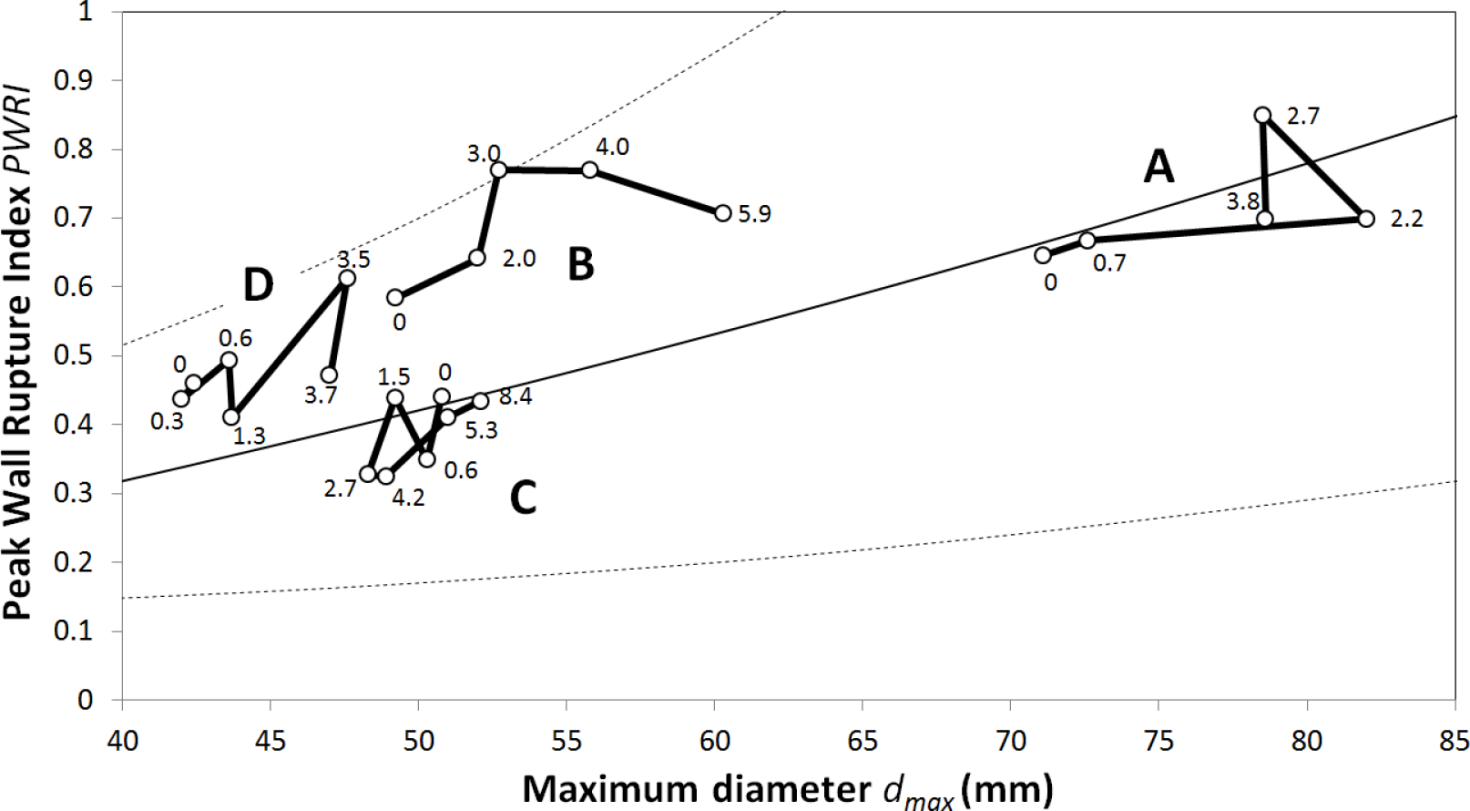
Development of the maximum diameter *d*_max_ and the Peak Wall Rupture Index (*PWRI*) in Abdominal Aortic Aneurysm (AAA) patients A to D. Each time point is labeled with the time in years from baseline. For comparison, the black solid curve denotes the *PWRI* versus *d*_max_ characteristics that in average is seen in AAA patients. Dotted curves denote the 5% and 95% confidence intervals, respectively.

*PWRI* and *d*_max_ varied considerably over time (Fig 4). AAA C is rather stable and slightly below the mean *PWRI* versus diameter curve. At baseline, AAA B has a slightly smaller diameter than AAA C (49mm versus 52mm) but a higher *PWRI*, and within 5.9 years its diameter grows up to 60mm. Interestingly, *PWRI* increases rapidly at first but slightly decreases later. Case D is rather small at baseline (42mm) at a *PWRI* between the cases B and C. After 3.5 years the diameter in case D reaches 48 mm, but subsequently both diameter and *PWRI* reduce. AAA A is already large at baseline (71 mm), and within 2.2 years its diameter grows to 82 mm, subsequently shrinking by about 4 mm.

### Correlation analysis

#### Simple correlation analysis

**Fig 5:**
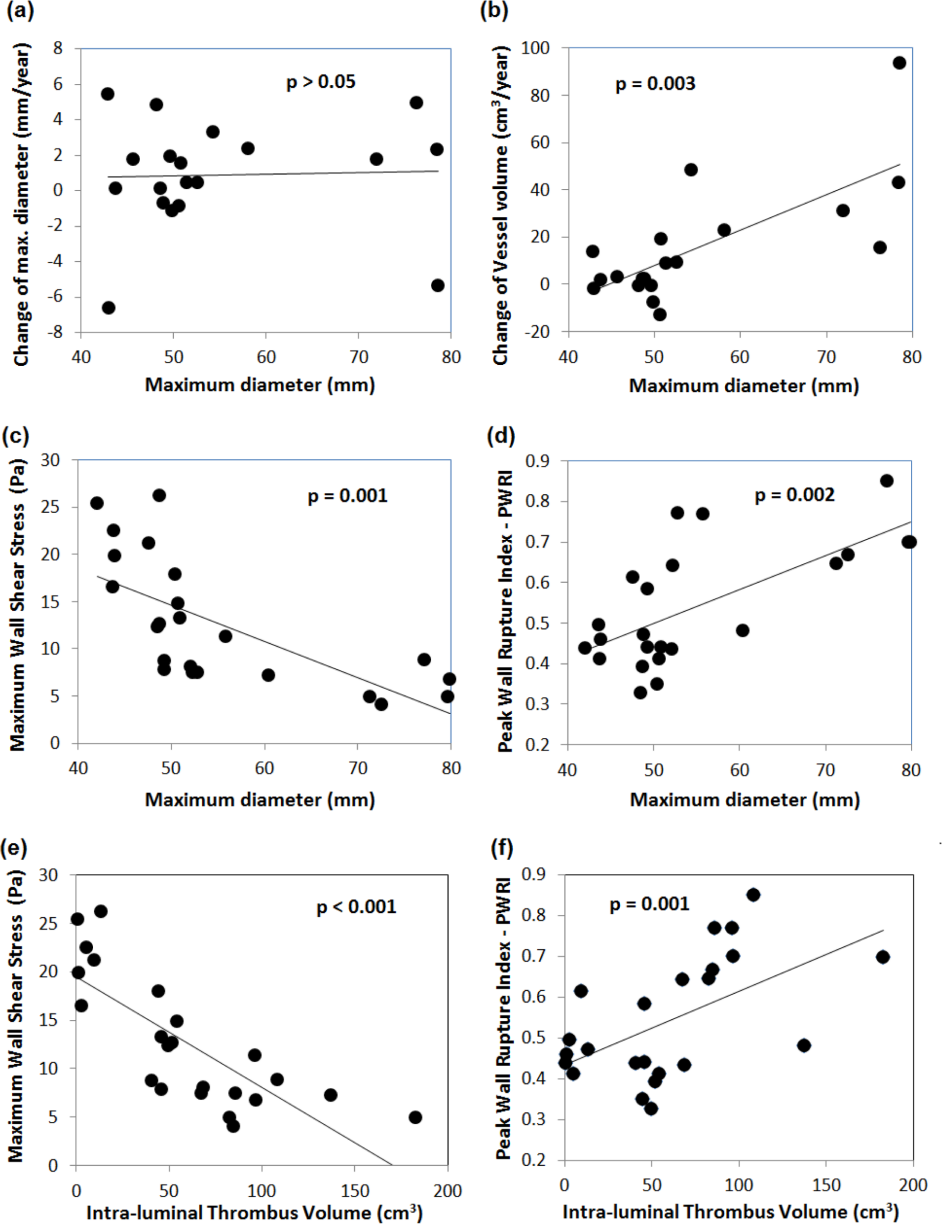
Influence of the maximum diameter on (a) the diameter growth Δ*d*_max_, (b) volume growth Δ*V*_tot_, (c) maximum Wall Shear Stress WSS_max_ over the cardiac cycle, and (d) Peak Wall Rupture Index PWRI at Mean Arterial Pressure (MAP). Influence of the Intra-luminal Thrombus (ILT) volume on (e) WSS_max_ over the cardiac cycle, and (f) PWRI at MAP.

Tables 3–6 summarize the results from the simple correlation analysis, and Fig 5A–5D illustrates key findings with respect to *d*_max_. Interestingly, *d*_max_ did not correlate with diameter growth Δ*d*_max_ (Fig 4A). Instead *d*_max_ correlated with volume growth Δ*V*_tot_, wall shear stress *WSS*_max_, and the biomechanical risk index *PWRI* (Fig 5B–5D). Moreover, trivial correlations between the diameter and volumes (*V*_*lum*_, *V*_tot_ and *V*_ILT_) were found.

**Table 3:** Correlations of geometrical and biomechanical parameters with the maximum diameter *d*_max_ (results are based on simple correlation analysis).

	Correlation coefficient	*p*-value
***H***_**ILT max**_	0.755	<0.001
***V***_**lum**_**; *V***_**tot**_**; *V***_**ILT**_	0.968; 0.936; 0.822	<0.001; <0.001; <0.001
***PWS***	0.891	<0.001
***PWRI***	0.672	0.002
${\dot{\gamma }}_{min};\;{\dot{\gamma }}_{mean}$	-0.773; -0.554	<0.0010.014
**WSS**_**max**_**; WSS**_**mean**_	-0.698; -0.459	0.001; 0.048
***OSI***	0.768	<0.001
***v***_**min**_**; *v***_**mean**_	-0.695; -0.519	<0.001; 0.023
**Δ*V***_**tot**_**; Δ*V***_**ILT**_	0.646; 0.501	0.003; 0.029

**Table 4:** Correlations of geometrical and biomechanical parameters with the ILT volume *V*_ILT_ (results are based on simple correlation analysis).

	Correlation coefficient	*p*-value
***H***_**ILT max**_	0.964	<0.001
***V***_**lum**_**; *V***_**tot**_	0.804; 0.941	<0.001; <0.001
***PWS***	0.640	0.003
***PWRI***	0.693	0.001
${\dot{\gamma }}_{min};\;{\dot{\gamma }}_{mean}$	-0.866; -0.580	<0.001; 0.009
**WSS**_**max**_**; WSS**_**mean**_	-0.829; -0.559	<0.001; 0.013
***OSI***	0.518	0.023
***v***_**mean**_	-0.584	0.009
**Δ*V***_**tot**_**; Δ*V***_**ILT**_	0.750; 0.605	<0.001; 0.006

**Table 5:** Correlations of geometrical and biomechanical parameters with the change of AAA volume Δ*V*_tot_ over time (results are based on simple correlation analysis).

	Correlation coefficient	*p*-value
***H***_**ILT max**_	0.804	<0.001
***V***_**lum**_**; *V***_**tot**_**; *V***_**ILT**_	0.697; 0.773; 0.750	0.001; <0.001; <0.001
***PWS***	0.584	0.009
***PWRI***	0.799	<0.001
${\dot{\gamma }}_{min}$	-0.615	0.005
**WSS**_**max**_	-0.577	0.010
***OSI***	0.475	0.040
***v***_**min**_	-0.477	0.039
**Δ*V***_**ILT**_	0.694	0.001

**Table 6:** Correlations of geometrical and biomechanical parameters with the change of ILT volume Δ*V*_ILT_ over time (results are based on simple correlation analysis).

	Correlation coefficient	*p*-value
***H***_**ILT max**_	0.627	0.004
***V***_**lum**_**; *V***_**tot**_**; *V***_**ILT**_	0.625; 0.666; 0.605	0.004; 0.002; 0.006
***PWS***	0.524	0.021
***PWRI***	0.696	0.001
${\dot{\gamma }}_{max};\;{\dot{\gamma }}_{min}$	0.548; -0.471	0.015; 0.042
***v***_**max**_	0.734	<0.001
**Δ*V***_**tot**_	0.694	0.001

The scalar shear rates ${\dot{\gamma }}_{min}$ and ${\dot{\gamma }}_{mean}$ as well as the wall shear stresses *WSS*_max_ (Fig 5E) and *WSS*_mean_ correlated negatively with *V*_ILT_. In contrast, the biomechanical risk index *PWRI* (Fig 5F) and the Oscillatory Shear Index *OSI* showed positive correlations with *V*_ILT_. In addition, the mean blood flow velocity *v*_mean_ correlated negatively with *V*_ILT_.

**Fig 6:**
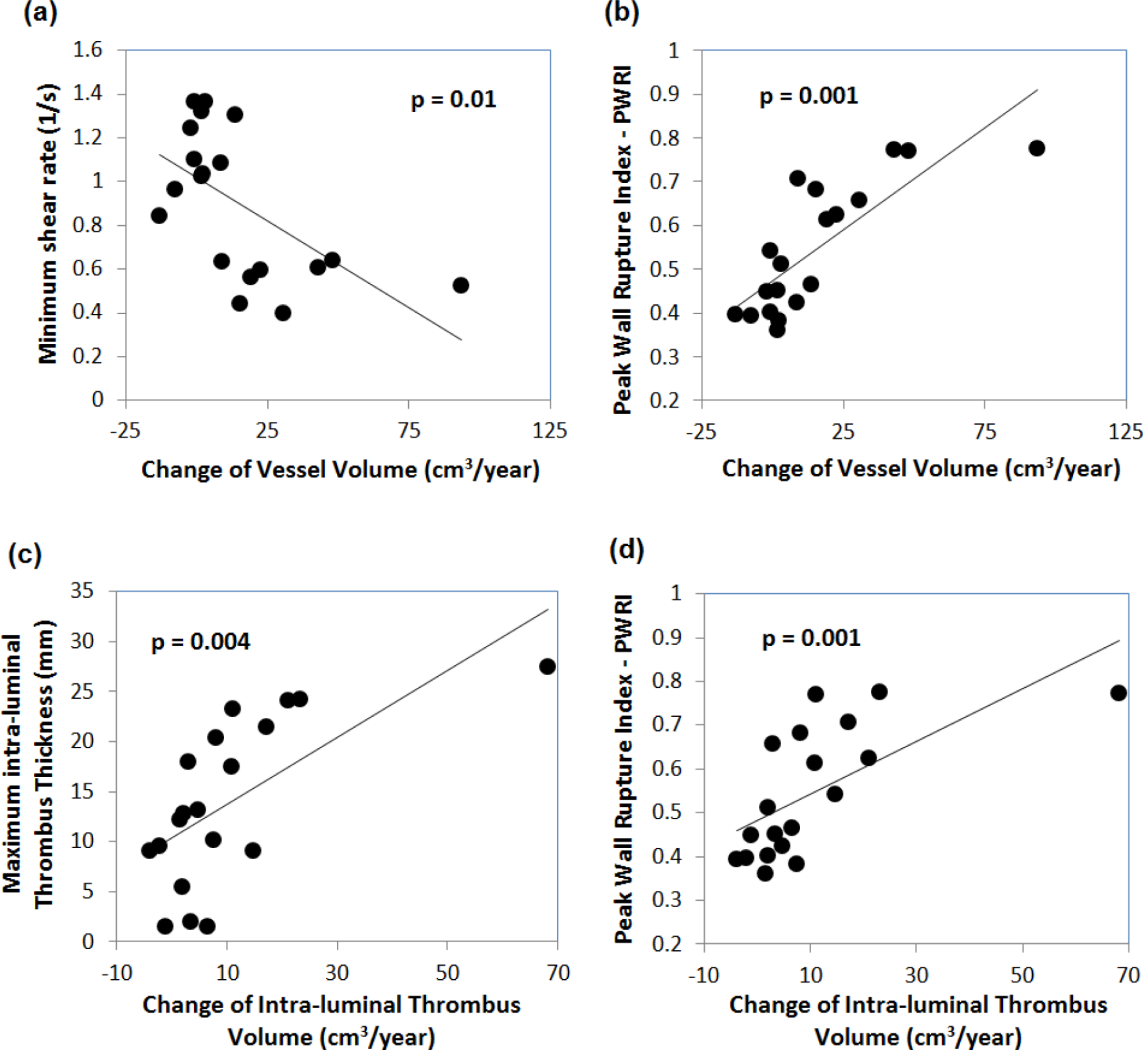
Influence of the change of vessel volume Δ*V*_tot_ on (a) the Minimum shear rate ${\dot{\gamma }}_{min}$ over the cardiac cycle, (b) Peak Wall Rupture Index PWRI at Mean Arterial Pressure (MAP). Influence of the Intra-Luminal Thrombus (ILT) volume growth rate Δ*V*_ILT_ on (c) maximum thickness of the ILT layer *H*_ILT max_, and (d) PWRI at MAP.

With respect to growth parameters, the maximum ILT thickness *H*_ILT max_ correlated with total volume growth Δ*V*_tot_ (Fig 6C). In addition, *PWRI* (Fig 6B) and *OSI* correlated positively, while ${\dot{\gamma }}_{min}$ (Fig 6A) correlated negatively with Δ*V*_tot_. Finally, simple regression with respect to the ILT growth Δ*V*_ILT_, exhibited correlations with *v*_max_, *PWRI* (Fig 6D), *H*_ILT max_ (Fig 6C) and ${\dot{\gamma }}_{max}$ (Table 3).

All identified correlations are given in the supporting information section.

#### Multiple correlation analysis

Multiple linear regression showed that both *WSS*_max_ (p = 0.004) and *PWRI* (p = 0.001) are independent predictors of vessel volume growth. Specifically, volume growth increased with low WSS_max_ and high PWRI following the relation Δ*V*_tot_ = a_0_ + a_1_*WSS*_max_ + a_2_*PWRI* with parameters a_0_ = −47.2 (CI_90%_: −89.4/−5.0), a_1_ = −0.411 (CI_90%)_: −1.713/0.892) and a_2_ = 124.1 (CI_90%)_: 69.4/178.7), where CI_90%_ denotes the 90% confidence interval.

Similarly, high *WSS*_max_ (p = 0.023) and *V*_ILT_ (p<0.001) independently predicted ILT volume growth according to Δ*V*_ILT_ = b_0_ + b_1_*WSS*_max_ + b_2_*V*_ILT_ with the parameters b_0_ = −48.38(CI_90%_: −75.73/−21.03), b_1_ = 2.169(CI_90%_: 0.859/3.479) and b_2_ = 0.541(CI_90%_: 0.346/0.736), respectively.

## Discussion

Clinical and experimental observations have indicated that biomechanical conditions influence the progression of aneurysm disease [45,46]. Despite these observations, a fundamental understanding of these interactions is still missing, particularly the role of the ILT in AAA pathology [25] is controversially discussed. The ILT is an active biochemical entity [25] that influences wall strength [12,24] and AAA progression [30], but also mechanically unloads the stress in the wall [20–22]. Specifically, clinical studies have linked a thick ILT layer [28] and fast increase in ILT volume [29] to increased AAA rupture risk. The present biomechanical study supports these observations through a strong positive correlation of the biomechanical risk index *PWRI* with both ILT volume *V*_ILT_ and its change over time Δ*V*_ILT_. Consequently, the suitability of monitoring ILT volume, and its change over time, as additional risk indicators should be explored in larger clinical studies.

ILT formation requires platelet accumulation, and for platelets to be able to adhere to the vessel, platelets must spend sufficient time in the vicinity of thrombogenic surfaces. Therefore, the adhesion of platelets might be promoted at sites of low *WSS* [43], i.e. an inverse relationship between *WSS* and aneurysm expansion may exist. Such an inverse relationship is confirmed by our study through the negative correlation of Δ*V*_tot_ with *WSS*. Similar conclusions have been drawn from clinical observations, experimental AAA models [46], and simulation studies [31]

The present study found that *PWRI* and WSS_max_ independently predicted the growth of total AAA volume Δ*V*_tot_. *PWRI* is strongly related to the stress in the wall, and our finding is supported by previous experimental studies [30] showing that the growth of small AAAs is especially sensitive to wall stress. Due to the lack of endothelial cells in AAAs [28], blood flow properties may only indirectly promote AAA growth through stimulation of the biochemical environment within the ILT. For example, a high *OSI* could support pumping proteolytic agents through the porous ILT, which in turn could promote AAA growth.

Contrary to intuition, our data showed that the biomechanical risk does not always increase in time. Wall stress is strongly linked to AAA shape parameters like its asymmetry [47] or, more generally, to the surface curvatures [41]. Consequently, if growth appears to reduce AAA asymmetry, the biomechanical risk for rupture also reduces, i.e. the aneurysm grows into a shape of lower risk for rupture. The fluctuations in PWRI could also be explained by releasing spots of high surface curvatures of the wall through “cracking” of wall calcifications during AAA expansion, for example.

The present study has several limitations. First of all our study was based on a relatively small number of cases due to the requirement of analyzing at least five CT-A scans for each patient. CT-A exposes patients to ionizing radiation and nephrotoxic contrast agents and should not be performed frequently. However, CT-A is practically the only standard image modality providing images accurate enough to build robust computational AAA models. Another limitation is related to the quantification of aneurysm growth. AAA growth is complex, and single parameters like change in maximum diameter or aneurysm volume can only serve as surrogate growth parameters. Therefore, a more rigorous three-dimensional quantification of the changing geometry would have been advantageous. However, CT-A images do not provide enough tracers in the wall that can be correlated amongst the different time points for a robust extraction of local wall growth. Such approach requires always some algorithms that interpolate between a few tracers (like anatomical landmarks) [48], and the extracted growth would always be largely influenced by algorithmic parameters, i.e. how the wall motion is interpolated between such tracers.

Biomechanical models introduce numerous modeling assumptions and cannot (and should not) completely reflect biomechanics of the real aneurysm. The constitution of aneurysm tissue and blood was modelled using mean population data. Patient-specific tissue and blood properties would have likely increased the accuracy of the predictions. Using a predefined AAA wall thickness influences wall stress predictions as well as ILT thickness measurements, and prescribing an inflow velocity profile influences blood flow predictions. Despite some of this information could be measured in the individual patient, the need for doing so remains unclear, and more research would be required to explore the sensitivity of our study results to such modeling assumptions. However, as these assumptions were used consistently across all patients they might not influence our conclusions.

## Supporting information

S1 Table Simple correlation analysis. Correlations amongst geometry, wall stress-related and blood flow-related biomechanical properties. ILTTmax- Maximum thickness of the Intra-Luminal Thrombus (ILT) layer; VILT–ILT volume; PWRI–Peak Wall Rupture Risk; RRED–Rupture Risk Equivalent Diameter; WSSmax—Maximal magnitude of the Wall Shear Stress (WSS) vector; WSSmin—Minimum magnitude of the WSS vector; WSSmean—Mean magnitude of the WSS vector; OSI—Oscillatory Shear Index; Velmax—Maximal magnitude of the blood flow velocity; Velmin—Minimal magnitude of the blood flow velocity; Velmean—Mean magnitude of the blood flow velocity; Shearmax—Maximal scalar shear rate; Shearmin—Minimal scalar shear rates; Shearmean—Mean scalar shear rate; dVlum–Increment in lumen volume; dVtot–Increment in total volume; dVILT–Increment in VILT; dPWRI–Increment in PWRI.
(XLS)

## References

[1] Choke E, Cockerill G, Wilson WRW, Sayed S, Dawson J, Loftus I, et al A review of biological factors implicated in abdominal aortic aneurysm rupture. Eur J Vasc Endovasc Surg. 2005;30: 227–244. doi: 10.1016/j.ejvs.2005.03.009 15893484 10.1016/j.ejvs.2005.03.009

[2] UK Small Aneurysm Trial Participants. Mortality results for randomised controlled trial of early elective surgery or ultrasonographic surveillance for small abdominal aortic aneurysms. Lancet. 1998;352: 1649–55. doi: 10.1016/S0140-6736(98)10137-X 9853436

[3] Greenhalgh Roger M., Powell Janet T. Endovascular repair of abdominal aortic aneurysm. N Engl J Med. 2010;363: 1480–1482. doi: 10.1002/14651858.CD004178.pub2 20931722 10.1056/NEJMc1007807

[4] Wanhainen A, Mani K, Golledge J. Surrogate Markers of Abdominal Aortic Aneurysm Progression. Arter Thromb Vasc Biol. 2016;36: 236–244. doi: 10.1161/ATVBAHA.115.306538 26715680 10.1161/ATVBAHA.115.306538

[5] Brown LC, Powell JT. Risk factors for aneurysm rupture in patients kept under ultrasound surveillance. UK Small Aneurysm Trial Participants. Ann Surg. 1999;230: 287–289. doi: 10.1097/00000658-199909000-00002 10.1097/00000658-199909000-00002 PMC1420874 10493476

[6] Darling RC, Messina CR, Brewster DC, Ottinger LW. Autopsy study of unoperated abdominal aortic aneurysms. Circulation. 1977;56: 161–164. 884821

[7] Nicholls SC, Gardner JB, Meissner MH, Johansen KH. Rupture in small abdominal aortic aneurysms. J Vasc Surg. 1998;28: 884–888. doi: 10.1016/S0741-5214(98)70065-5 9808857 10.1016/s0741-5214(98)70065-5

[8] Choksy SA, Wilmink AB, Quick CR. Ruptured abdominal aortic aneurysm in the Huntingdon district: a 10-year experience. Ann R Coll Surg Engl. 1999;81: 27–31. Available: http://www.pubmedcentral.nih.gov/articlerender.fcgi?artid=2503249&tool=pmcentrez&rendertype=abstract 10325681 PMC2503249

[9] Fillinger MF, Raghavan ML, Marra SP, Cronenwett JL, Kennedy FE. In vivo analysis of mechanical wall stress and abdominal aortic aneurysm rupture risk. J Vasc Surg. 2002;36: 589–597. doi: 10.1067/mva.2002.125478 12218986 10.1067/mva.2002.125478

[10] Fillinger MF, Marra SP, Raghavan ML, Kennedy FE. Prediction of rupture risk in abdominal aortic aneurysm during observation: Wall stress versus diameter. J Vasc Surg. 2003;37: 724–732. doi: 10.1067/mva.2003.213 12663969 10.1067/mva.2003.213

[11] Venkatasubramaniam AK, Fagan MJ, Mehta T, Mylankal KJ, Ray B, Kuhan G, et al A comparative study of aortic wall stress using finite element analysis for ruptured and non-ruptured abdominal aortic aneurysms. Eur J Vasc Endovasc Surg. 2004;28: 168–176. doi: 10.1016/j.ejvs.2004.03.029 15234698 10.1016/j.ejvs.2004.03.029

[12] Vande Geest JP, Di Martino ES, Bohra A, Makaroun MS, Vorp DA. A biomechanics-based rupture potential index for abdominal aortic aneurysm risk assessment: Demonstrative application. Ann N Y Acad Sci. 2006;1085: 11–21. doi: 10.1196/annals.1383.046 17182918 10.1196/annals.1383.046

[13] Truijers M, Pol JA, SchultzeKool LJ, van Sterkenburg SM, Fillinger MF, Blankensteijn JD. Wall Stress Analysis in Small Asymptomatic, Symptomatic and Ruptured Abdominal Aortic Aneurysms. Eur J Vasc Endovasc Surg. 2007;33: 401–407. doi: 10.1016/j.ejvs.2006.10.009 17137809 10.1016/j.ejvs.2006.10.009

[14] Geest JP Vande, Schmidt DE, Sacks MS, David A. The Effects of Anisotropy on the Stress Analyses of Patient- Specific Abdominal Aortic Aneurysms. 2008;36: 921–932. doi: 10.1007/s10439-008-9490-3.The 10.1007/s10439-008-9490-3 PMC2674610 18398680

[15] Maier A, Gee MW, Reeps C, Pongratz J, Eckstein HH, Wall WA. A comparison of diameter, wall stress, and rupture potential index for abdominal aortic aneurysm rupture risk prediction. Ann Biomed Eng. 2010;38: 3124–3134. doi: 10.1007/s10439-010-0067-6 20480238 10.1007/s10439-010-0067-6

[16] Gasser TC, Nchimi A, Swedenborg J, Roy J, Sakalihasan N, Böckler D, et al A novel strategy to translate the biomechanical rupture risk of abdominal aortic aneurysms to their equivalent diameter risk: Method and retrospective validation. Eur J Vasc Endovasc Surg. Elsevier Ltd; 2014;47: 288–295. doi: 10.1016/j.ejvs.2013.12.018 24456739 10.1016/j.ejvs.2013.12.018

[17] McGloughlin TM, Doyle BJ. New approaches to abdominal aortic aneurysm rupture risk assessment: Engineering insights with clinical gain. Arterioscler Thromb Vasc Biol. 2010;30: 1687–1694. doi: 10.1161/ATVBAHA.110.204529 20508202 10.1161/ATVBAHA.110.204529

[18] Gasser TC, Auer M, Labruto F, Swedenborg J, Roy J. Biomechanical rupture risk assessment of abdominal aortic aneurysms: Model complexity versus predictability of finite element simulations. Eur J Vasc Endovasc Surg. Elsevier Ltd; 2010;40: 176–185. doi: 10.1016/j.ejvs.2010.04.003 20447844 10.1016/j.ejvs.2010.04.003

[19] Hans SS, Jareunpoon O, Balasubramaniam M, Zelenock GB. Size and location of thrombus in intact and ruptured abdominal aortic aneurysms. J Vasc Surg. 2005;41: 584–588. doi: 10.1016/j.jvs.2005.01.004 15874920 10.1016/j.jvs.2005.01.004

[20] Mower WR, J. QW, Gambhir SS. Effect of Intraluminal Thrombus on Local Abdominal Aortic Aneurysm Wall Stress. Proc first Jt BMES/EMBS Conf. 1997;27: 244 doi: 10.1016/S0741-5214(97)70058-2 10.1016/s0741-5214(97)70058-2 9357460

[21] Li ZY, U-King-Im J, Tang TY, Soh E, See TC, Gillard JH. Impact of calcification and intraluminal thrombus on the computed wall stresses of abdominal aortic aneurysm. J Vasc Surg. 2008;47: 928–935. doi: 10.1016/j.jvs.2008.01.006 18372154 10.1016/j.jvs.2008.01.006

[22] Thubrikar MJ, Robicsek F, Labrosse M, Chervenkoff V, Fowler BL. Effect of thrombus on abdominal aortic aneurysm wall dilation and stress. J Cardiovasc Surg (Torino). 2003;44: 67–77. 12627076

[23] Riveros F, Martufi G, Gasser TC, Rodriguez-Matas JF. On the Impact of Intraluminal Thrombus Mechanical Behavior in AAA Passive Mechanics. Ann Biomed Eng. 2015;43: 2253–2264. doi: 10.1007/s10439-015-1267-x 25636600 10.1007/s10439-015-1267-x

[24] Vorp DA, Lee PC, Wang DHJ, Makaroun MS, Nemoto EM, Ogawa S, et al Association of intraluminal thrombus in abdominal aortic aneurysm with local hypoxia and wall weakening. J Vasc Surg. 2001;34: 291–299. doi: 10.1067/mva.2001.114813 11496282 10.1067/mva.2001.114813

[25] Swedenborg J, Eriksson P. The intraluminal thrombus as a source of proteolytic activity. Ann N Y Acad Sci. 2006;1085: 133–138. doi: 10.1196/annals.1383.044 17182929 10.1196/annals.1383.044

[26] Adolph R, Vorp DA, Steed DL, Webster MW, Kameneva M V., Watkins SC. Cellular content and permeability of intraluminal thrombus in abdominal aortic aneurysm. J Vasc Surg. 1997;25: 916–926. doi: 10.1016/S0741-5214(97)70223-4 9152321 10.1016/s0741-5214(97)70223-4

[27] Gasser TC, Görgülü G, Folkesson M, Swedenborg J. Failure properties of intraluminal thrombus in abdominal aortic aneurysm under static and pulsating mechanical loads. J Vasc Surg. 2008;48: 179–188. doi: 10.1016/j.jvs.2008.01.036 18486417 10.1016/j.jvs.2008.01.036

[28] Kazi M, Thyberg J, Religa P, Roy J, Eriksson P, Hedin U, et al Influence of intraluminal thrombus on structural and cellular composition of abdominal aortic aneurysm wall. J Vasc Surg. 2003;38: 1283–1292. doi: 10.1016/S0741 14681629 10.1016/s0741-5214(03)00791-2

[29] Stenbaek J, Kalin B, Swedenborg J. Growth of thrombus may be a better predictor of rupture than diameter in patients with abdominal aortic aneurysms. Eur J Vasc Endovasc Surg. 2000;20: 466–469. doi: 10.1053/ejvs.2000.1217 11112467 10.1053/ejvs.2000.1217

[30] Martufi G, Lindquist Liljeqvist M, Sakalihasan N, Panuccio G, Hultgren R, Roy J, et al Local Diameter, Wall Stress and Thrombus Thickness Influence the Local Growth of Abdominal Aortic Aneurysms. Eur J Vasc Endovasc Surg. 2014;48: 349 doi: 10.1016/j.ejvs.2014.06.032 10.1177/1526602816657086 27412646

[31] Zambrano BA, Gharahi H, Lim C, Jaberi FA, Lee W, Baek S, et al Association of intraluminal thrombus, hemodynamic forces, and abdominal aortic aneurysm expansion using longitudinal CT images. Ann Biomed Eng. 2016;44: 1502–1514. doi: 10.1007/s10439-015-1461-x 26429788 10.1007/s10439-015-1461-x PMC4826625

[32] Gasser TC. Biomechanical Rupture Risk Assessment: A Consistent and Objective Decision-Making Tool for Abdominal Aortic Aneurysm Patients. Aorta (Stamford, Conn). 2016;4: 42–60. doi: 10.12945/j.aorta.2015.15.030 27757402 10.12945/j.aorta.2015.15.030 PMC5054755

[33] Hyhlik-Dürr A, Krieger T, Geisbüsch P, Kotelis D, Able T, Böckler D. Reproducibility of Aortic Diameter, Volume, Peak Wall Stress, and Peak Rupture Risk Index Using Semiautomatic Finite Element Analyses of Infrarenal Aortic Aneurysms. J Endovasc Ther. 2011;18: 289–298. doi: 10.1583/10-3384MR.1 21679063 10.1583/10-3384MR.1

[34] Auer M, Gasser TC. Reconstruction and Finite Element Mesh Generation of Abdominal Aortic Aneurysms From Computerized Tomography Angiography Data With Minimal User Interactions. IEEE Trans Med Imaging. 2010;29: 1022–1028. doi: 10.1109/TMI.2009.2039579 20335091 10.1109/TMI.2009.2039579

[35] Teutelink A, Cancrinus E, Van De Heuvel D, Moll F, De Vries JP. Preliminary intraobserver and interobserver variability in wall stress and rupture risk assessment of abdominal aortic aneurysms using a semiautomatic finite element model. J Vasc Surg. Elsevier Inc.; 2012;55: 326–330. doi: 10.1016/j.jvs.2011.08.012 22104340 10.1016/j.jvs.2011.08.012

[36] Biasetti J, Gasser TC, Auer M, Hedin U, Labruto F. Hemodynamics of the normal aorta compared to fusiform and saccular abdominal aortic aneurysms with emphasis on a potential thrombus formation mechanism. Ann Biomed Eng. 2010;38: 380–390. doi: 10.1007/s10439-009-9843-6 19936925 10.1007/s10439-009-9843-6

[37] Mills C. J., Gabe I. T. JHG, Mason D. T., Ross J J., Braunwald E., Shillingford J. P. Pressure-flow relationships and vascular impedance in man. Cardiovasc Res. 1970;4: 405–417. 5533085 10.1093/cvr/4.4.405

[38] Simo JC, Taylor RL. Quasi-incompressible finite elasticity in principal stretches. continuum basis and numerical algorithms. Comput Methods Appl Mech Eng. 1991;85: 273–310. doi: 10.1016/0045-7825(91)90100-K

[39] Raghavan ML, Vorp DA. Toward a biomechanical tool to evaluate rupture potential of abdominal aortic aneurysm: Identification of a finite strain constitutive model and evaluation of its applicability. J Biomech. 2000;33: 475–482. doi: 10.1016/S0021-9290(99)00201-8 10768396 10.1016/s0021-9290(99)00201-8

[40] Prakash S, Ethier CR. Requirements for mesh resolution in 3D computational hemodynamics. J Biomech Eng. 2001;123: 134–144. doi: 10.1115/1.1351807 11340874 10.1115/1.1351807

[41] Leea Kibaek, Zhua Junjun, Shumb Judy, Zhanga Yongjie, Mulukc Satish C., Chandrad Ankur, et al Surface Curvature as a Classifier of Abdominal Aortic Aneurysms: A Comparative Analysis. Ann Biomed Eng. 2012;100: 130–134. doi: 10.1016/j.pestbp.2011.02.012.Investigations 10.1007/s10439-012-0691-4 PMC3600104 23180028

[42] Leuprecht A, Perktold K. Computer simulation of non-newtonian effects on blood flow in large arteries. Comput Methods Biomech Biomed Engin. 2001;4: 149–163. doi: 10.1080/10255840008908002 11264865 10.1080/10255840008908002

[43] Biasetti J, Hussain F, Gasser TC. Blood flow and coherent vortices in the normal and aneurysmatic aortas: a fluid dynamical approach to intra-luminal thrombus formation. J R Soc Interface. 2011;8: 1449–1461. doi: 10.1098/rsif.2011.0041 21471188 10.1098/rsif.2011.0041 PMC3163425

[44] Ku DN, Giddens DP, Zarins CK, Glagov S. Pulsatile flow and atherosclerosis in the human carotid bifurcation. Positive correlation between plaque location and low oscillating shear stress. Arterioscler Thromb Vasc Biol. 1985;5: 293–302. doi: 10.1161/01.ATV.5.3.293 10.1161/01.atv.5.3.293 3994585

[45] Bäck M, Gasser TC, Michel JB, Caligiuri G. Biomechanical factors in the biology of aortic wall and aortic valve diseases. Cardiovasc Res. 2013;99: 232–241. doi: 10.1093/cvr/cvt040 23459103 10.1093/cvr/cvt040 PMC3695745

[46] Dua MM, Dalman RL. Hemodynamic Influences on abdominal aortic aneurysm disease: Application of biomechanics to aneurysm pathophysiology. Vascul Pharmacol. 2010;53: 11–21. doi: 10.1016/j.vph.2010.03.004 20347049 10.1016/j.vph.2010.03.004 PMC2880166

[47] Vorp DA, Raghavan ML, Webster MW. Mechanical wall stress in abdominal aortic aneurysm: Influence of diameter and asymmetry. J Vasc Surg. 1998;27: 632–639. doi: 10.1016/S0741-5214(98)70227-7 9576075 10.1016/s0741-5214(98)70227-7

[48] Satriano A, Rivolo S, Martufi G, Finol EA, Di Martino ES. In vivo strain assessment of the abdominal aortic aneurysm. J Biomech. 2015;48: 354–360. doi: 10.1016/j.jbiomech.2014.11.016 25497379 10.1016/j.jbiomech.2014.11.016

